# Visualizing Dynamic Bitcoin Transaction Patterns

**DOI:** 10.1089/big.2015.0056

**Published:** 2016-06-01

**Authors:** Dan McGinn, David Birch, David Akroyd, Miguel Molina-Solana, Yike Guo, William J. Knottenbelt

**Affiliations:** Data Science Institute, Imperial College London, London, United Kingdom.

**Keywords:** big data analytics, bitcoin, cryptocurrency, large-scale graph visualization, money laundering, pattern recognition, structured data

## Abstract

This work presents a systemic top-down visualization of Bitcoin transaction activity to explore dynamically generated patterns of algorithmic behavior. Bitcoin dominates the cryptocurrency markets and presents researchers with a rich source of real-time transactional data. The pseudonymous yet public nature of the data presents opportunities for the discovery of human and algorithmic behavioral patterns of interest to many parties such as financial regulators, protocol designers, and security analysts. However, retaining visual fidelity to the underlying data to retain a fuller understanding of activity within the network remains challenging, particularly in real time. We expose an effective force-directed graph visualization employed in our large-scale data observation facility to accelerate this data exploration and derive useful insight among domain experts and the general public alike. The high-fidelity visualizations demonstrated in this article allowed for collaborative discovery of unexpected high frequency transaction patterns, including automated laundering operations, and the evolution of multiple distinct algorithmic denial of service attacks on the Bitcoin network.

## Introduction

Deriving insight into the dense data sets generated by modern computational and sensing systems is still primarily performed by humans in possession of domain knowledge and the necessary mathematical and statistical tools. Visualization has also been shown to be an effective way of gaining insights into the available data. In that regard, the volume edited by Card et al.^1^ is still a valuable reference and provides plenty of examples of such visualizations.

A system of interest, which generates a large amount of connected data and lacks meaningful systemic visualization tools, is that of Bitcoin.^2^ This cryptocurrency system is primarily composed of a permissionless public database to which anyone with a tokenized pseudonymous identity may write protocol-conformant data. Since identity is obfuscated through the use of tokenized addresses, the ability to identify and classify anomalous patterns of behavior in the data has utility to many interested parties such as financial regulators (e.g., in the case of money laundering activity) or protocol developers (in the case of attacks on the system's resilience). Conducting an initial graphical observation is a useful first step in the data-analysis workflow to investigate the structural properties of such repeated anomalous behaviors. We investigated different visualizations able to provide this useful exploratory insight into the underlying behaviors observable in the data.

This article describes the design and development of tools for dynamically visualizing Bitcoin transactions. The visualizations demonstrated in this article have enabled the discovery of unexpected transaction patterns such as money laundering activity and the observation of several distinct denial of service attacks on the Bitcoin network. This allowed rapid understanding among researchers of the structure of such behavioral patterns for accelerated analysis and classification investigation.

**FIG. 1.**
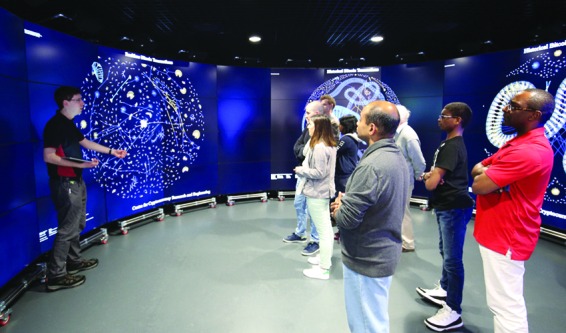
Bitcoin visualizations presented in a large-scale data observatory.

The tools have been successfully deployed in our data observatory facility:^3,4^ a high-resolution 64 screen distributed rendering cluster with a canvas of 132M pixels (Fig. 1). We reflect upon how the employment of such a large-scale observatory environment benefits more effective data visualization and provides for greater insight into the data.

## Bitcoin Network and Data

Bitcoin, with its inception dating from 2009, is the dominant cryptocurrency implementation. The system is primarily composed of an agreed protocol for broadcasting exchanges of value between tokenized participants of a peer-to-peer network. These *transaction* records are subsequently regularly verified by specialist “mining” nodes on the network, whose honesty is ensured through economic jeopardy, and recorded into a publicly distributed tamperproof ledger known as the *blockchain*. By design, this database and its updates are public to allow a real-time majority consensus to form as to the current valid system state. In this way, through the elegant coupling of cryptography with economic incentives, participating pseudonymous strangers are able to establish mutual trust and conduct secure transactions among themselves with high confidence.

The raw blockchain database by the end of 2015 stands at ∼50 GB and contains a continuous record of the initial minting of every amount of bitcoin and every subsequent transfer of ownership since the system's inception.

Within the Bitcoin network, several protocol conformant data structures are propagated around the peer-to-peer network using a gossip algorithm. The entire Bitcoin system exists exclusively to create, propagate, verify, and record data structures known as *transactions*. A transaction is an atomic record through which ownership of an amount of bitcoin is transferred by the current owner to a new owner. A transaction is composed of 1.*n outputs* and 0.*n inputs*. Transaction outputs are new records of amounts of bitcoin along with an associated encumbrance to a particular Bitcoin *address*, being a representation of the public key component of an asymmetric cryptographic challenge satisfiable only by the new owner. Transaction inputs are pointers to existing *unspent* transaction outputs (UTXO's) along with a valid proof of the particular UTXO's existing cryptographic challenge to verifiably demonstrate ownership. It is only through the provision of all the input solutions to the cryptographic challenges that a transaction will be recognized and recorded by participants as valid, preventing theft. Similarly any transaction attempting to reassign ownership of previously unencumbered amounts (double spend) or include outputs summing to more than the inputs (counterfeiting) will be rejected by the majority of honest participants. Each new transaction's unspent outputs can therefore be considered the frontier edge of a particular tree of spends through the entire transaction graph, rooted at a set of coinbase transactions.

Transactions are broadcast around the network and each participating node will keep a copy of received transactions that it considers valid in a data structure held in volatile memory known as the *mempool*.

Specialist nodes on the peer-to-peer network known as *miners* proceed to select a set of transactions of their choosing from their own mempool and package them into a data structure known as a *block*. By including a special reward to themselves known as a *coinbase* transaction, a miner will generate a block header summarizing this static transaction data set along with some metadata, including a reference to the previous valid block. The miner will then set about solving a variable nonce field in a sequential brute-force manner such that the block header's cryptographic fingerprint satisfies the current network-wide difficulty criteria.

Once a miner finds a winning solution to this lottery (whose difficulty is amended approximately every 2 weeks to result in an average block solution every 10 minutes and the probability of winning such is directly proportional to the amount of processing power invested in the lottery), the block is broadcast around the network to be checked by each node against a set of validation criteria. If the block and every transaction contained therein are conformant to the agreed protocol, each full node on the network will add the block to its own independent local copy of the blockchain. All miners will then commence a new race to solve a block of the next transaction set. Thus, a network-wide consensus on the valid system state is reached, and any node can recreate the current consensus system state independently.

By its nature, anyone participating in the network has access to all data in binary form through TCP connections to neighboring nodes. In generating our visualizations, however, we chose to use some of the many curated and generously free feeds from Bitcoin data providers, particularly Blockchain.info and Bitnodes.21.co, using standard RESTful technologies such as websockets and http requests.

## Previous Work and Design Motivations

The granular and public nature of the Bitcoin dataset presents a unique opportunity for the study of a closed economic system at such scale and has already attracted much analysis. Such analyses have typically focused on bottom-up approaches to deriving useful information from the Bitcoin system, either by analyzing individual address use in the blockchain and inferring clusterings of ownership/deanonymization^5–8^ or by relating individual transactions directly to infer some associated behaviors such as money laundering.^9,10^ The use of visualization thus far has been used to a limited extent solely to present the results of these bottom-up approaches. The first interesting deployment of small-scale visualization to directly analyze transaction data in the blockchain is presented by Di Battista et al.,^11^ which exposes a tool to perform a bottom-up visual analysis of the influence of selected source transactions on subsequent flows in the transaction graph.

With 132M pixels at our disposal, our motivation was to generate a top-down system-wide visualization to explain Bitcoin to a lay audience and begin an explorative analysis of algorithmic patterns of associated behaviors in the transaction data.

The Bitcoin blockchain, with its canonical ordering of sequences of transactions and associations between spending addresses, naturally lends itself to graph visualization and that is the focus of our work. However, faced with the large size of the full transaction graph described in Table 1, any visualization effort is forced to compromise between which discrete subset of data to visualize and how to abstract away unnecessary detail. Previous bottom-up approaches have achieved this by restricting the scope of their analyses to identifying a limited subset of starting points of interest in the blockchain from which to visualize. Address-based graph visualizations have typically been separated from transaction-based graphs. Furthermore, details of the particular associations in transaction graphs are usually abstracted away into summary form. Specifically a transaction is the only type of node represented in typical transaction graph visualizations, with its edge associations between its inputs and any number of other transactions and their outputs abstracted to a single-labeled edge between transaction nodes. While retaining enough information for quantitative analysis, the visual fidelity to the underlying data is much reduced. Concretely, visually identifying a transaction with an unusually large number of outputs or an anomalous amount of Bitcoin sourced from a previous transaction becomes an arduous visual operation on textual data in such abstracted form.

**Table 1.** **Bitcoin blockchain summary statistics at the 7th year anniversary of the genesis block on January 3, 2016**

Total bytes:	54,814,349,473
Total blocks:	391,570
Total transactions:	101,533,304
Total inputs:	267,860,693
Total outputs:	301,970,961
Implied UTXO's:	34,110,268
BTC minted:	15,039,250
Market capitalization @431$/B:	$6.48bn

BTC, bitcoin; UTXO, unspent transaction output.

**FIG. 2.**
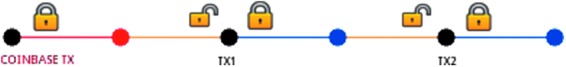
Visualizing a simple chain of spends in the mempool with blue outputs from one transaction becoming orange inputs to the next, from a source coinbase transaction in red.

With the full benefit of the large-scale digital canvas available in our data observatory, our visualization goal was to remain as faithful to the underlying data as possible to retain the richest observational insight into the identification of anomalies and patterns of behavior. In particular, we found it important to retain visual impact regarding the input and output structure of a transaction, the relative value of transactions, and to maintain associations between both transactions and addresses within the scope of a single visualization. We chose to restrict our subset of blockchain data based on sequential series of blocks without abstraction. To layout our graph in a force-directed minimum energy equilibrium state to visually discern its structure, we used the continuous ForceAtlas2^12^ algorithm available in the SigmaJS^13^ library. The implementation provides for Barnes–Hut optimization familiar to *n*-body simulations to reduce the computational complexity from *O*(*N*^2^) to *O*(*NlogN*). To that end, the basic design of our graph visualization is as follows:

• *Transactions* are visualized as nodes in a neutral color whose size is fixed at the value of the current coinbase reward (25BTC) to give a fixed sense of scale since the size of input and output nodes is variable depending on value. A transaction node's only purpose though is to provide a local focus for its associated inputs and outputs.
• *Inputs* are nodes of an orange color whose size is proportional to its value. They are associated to their containing transaction by an orange edge.
• *Outputs* are nodes blue in color whose size is also proportional to its value. They are associated to their containing transaction by a blue edge and if an output should become referenced as an input in a subsequent transaction within the scope of the visualization, it is joined to that transaction by an orange input edge, thus forming a chain of spends (Fig. 2).
• *Addresses* are visualized as a gray associative edge only if more than one input or output references the same address within the scope of the visualization.

**FIG. 3.**
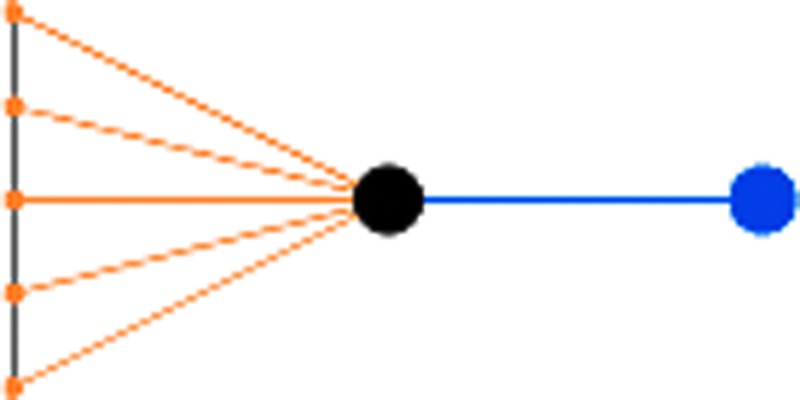
Stylized transaction visualization sourcing five equal input amounts from a single address and paying 25BTC to a new address.

It can be seen from the stylized representation shown in Figure 3 that all contextual and association information from the transaction data structure can be visualized in one graph and thus any amounts, structures of individual transactions, high-frequency chains of spends, or address associations of an anomalous nature will be immediately apparent by visual inspection.

## Visualizing Bitcoin Transactions

We now take our transaction representation and apply it to an animated graph whose layout evolves in real time to visualize transactions and their associations as they are broadcast into the network and join all peers' mempools. Furthermore, we apply the same animated force-directed visualization to explore individual blocks of static data laid out on request to explore past behaviors. To gently introduce a lay audience to some of the abstract concepts of Bitcoin, we also produced a global visual manifestation of the activity on the peer-to-peer network, less intimidating in its complexity.

## Mempool Visualization

**FIG. 4.**
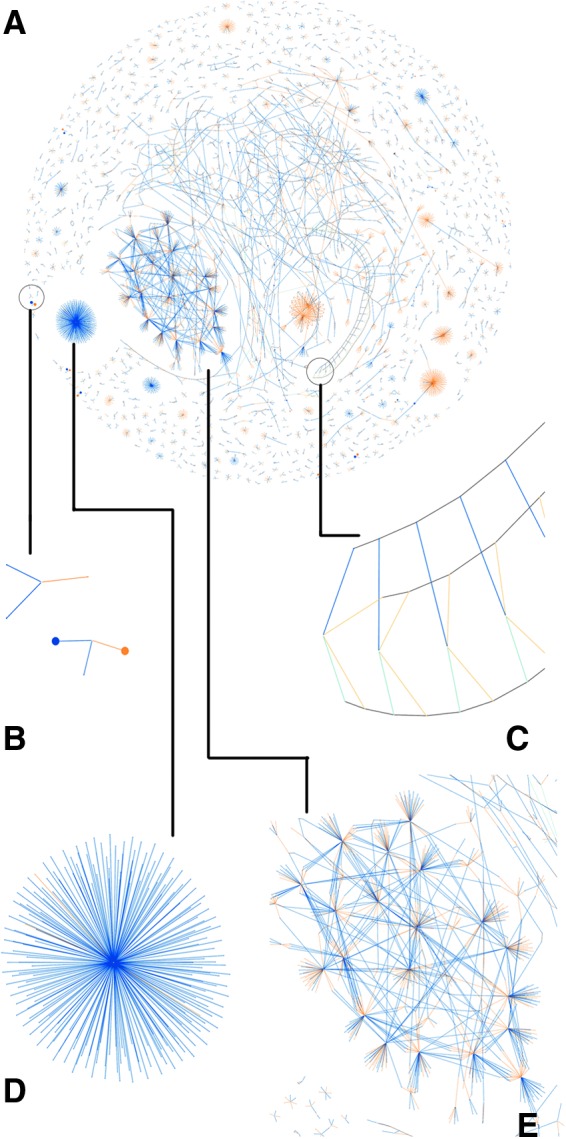
**(A)** High-resolution (8k) visualization of a standard block; **(B)** detail of both a low (small node) and a high (large node) value transaction, **(C)** known and linked Bitcoin addresses, **(D)** a payout system, and **(E)** a highly associated disconnected component believed to be a coin-tumbling service to move amounts rapidly between addresses, obfuscating the source and destination of funds.

The aim of this animated visualization^*^ (e.g., Fig. 4A) was to demonstrate the current activity and degree of connectivity as transactions enter the mempool in real time through a continuously updated force-directed graph layout. By interacting with the Bitcoin network through known stained addresses, it is also possible to conduct an active data analysis by identifying one's own transactions and the network's responses.

Independent transactions are visually associated to each other in two ways: either directly through an existing output becoming an input to a new transaction within the timeframe of the visualization or indirectly through the reuse of the same cryptographic public key within an element of a transaction, which we connect with a gray edge.

Interacting with the visualization is simple. We provide for pan, zoom, and hover over methods to display uncluttered textual data such as transaction references and address information. We facilitate further detailed data analysis by highlighting connected components along with the ability to transmit such subcomponent data in JSON by PeerJS to hand-held tablet displays for a more detailed, localized analysis directly linked to online Bitcoin exploration tools such as Blockchain.info. Filtering the visualized data set by amount, address, or reference is also possible from the hand-held tablet display.

The current Bitcoin transaction rate under normal circumstances is around 2–3 per second. A typical simple transaction, as shown in Figure 4B, will be rendered in our visualization with four vertices (the transaction, an input, a spending output, and an output back to the current owner for an amount of change). Considering more sophisticated transactions with many inputs and/or outputs, data are rendered into our graph at a rate of around 500–1000 new vertices per minute, allowing a manageable real-time layout and visually clear rendering using standard web technologies. This enables scalability to explore historical transactions.

We store an index of the 2000 latest transactions in a circular buffer, which when full removes the oldest transactions from the visualization on a First-In–First-Out basis. Transactions are also removed from this visualization should they be included in any block as it is broadcast into the network. In this way, computational load in rendering the layout is continuously managed such that the number of nodes in the visualization is never more than around 10,000 (given the multiple inputs and outputs associated with each transaction).

## Blockchain Visualization

**FIG. 5.**
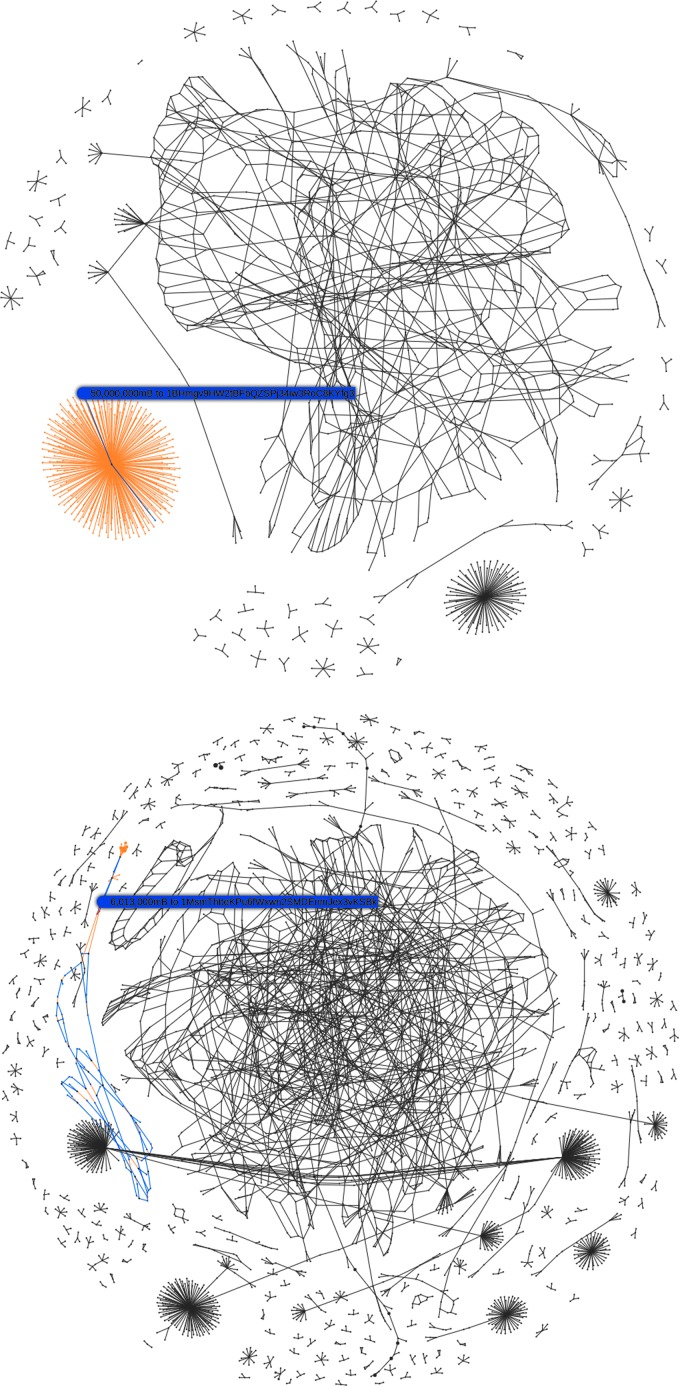
Visualizing blocks#199884,232304, previously reported as containing anomalous yet unidentified transactions at the apex of a money laundering operation,^21^ demonstrating ease of visual search and hover-over interaction for isolation and further analysis.

This visualization is similar in nature to the mempool, but provides the ability to visually explore any individual block mined into the blockchain. It allows the visual recognition of recurring patterns within the average 10-minute timeframe of a block. Examples of this visualization are shown in Figures 4 and 5. Special coinbase transactions rewarding miners (which are not broadcast in the network and thus inapplicable to the mempool visualization) have no source inputs since they are newly minted coins and are visualized here in red.

Expanded later in this article, this visualization has allowed us to detect anomalous high-frequency behavioral patterns within the Bitcoin transaction graph and demarcate a period of artificial network stress into two distinct and independent behaviors that were previously hidden in the dense raw dataset.

Building on previous analysis,^10^ section 3.5 “Use Cases” of Di Battista et al.^11^ used visualization to reveal two anomalous transactions at the apex of a money laundering operation, but did not identify them by reference. Figure 5 shows the ease with which our tool allows immediate visual identification of these transactions, given knowledge only of their anomalous nature.

## Peer Visualization

**FIG. 6.**
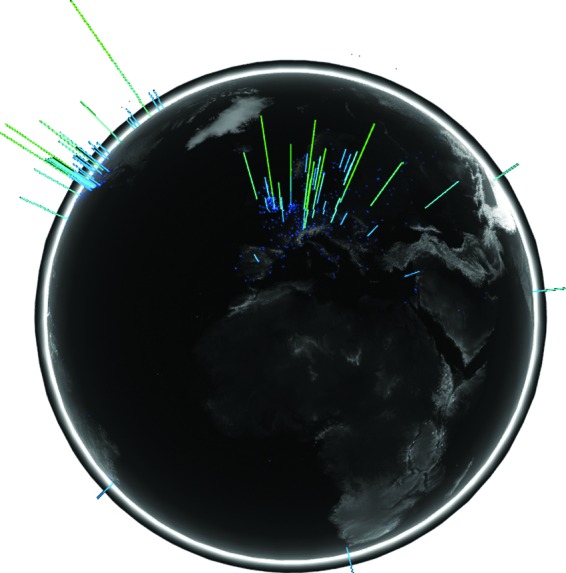
Global visualization of contactable nodes and transaction activity on the Bitcoin peer-to-peer network.

The aim of this simple rotating globe visualization, shown in Figure 6, was to demonstrate the global scope of the peer-to-peer network and bring to life areas of activity. Knowledge of network topology is not only important to ensure network robustness and efficient data propagation but also to determine which nodes may have an advantage and which attacks on the system may be feasible.^14,15^

A Bitcoin Core node cold booting into the P2P network embarks on a process of network discovery through the use of hardcoded DNS servers; it subsequently maintains knowledge of up to 2000 peers in its local addrMan database through the gossip of ADDR messages despite only initiating a maximum of eight actual peer connections.

The vast majority of peers on the network are behind firewalls/NATs, and therefore maintain their network presence solely through their eight outgoing connections alone, while rejecting all incoming connection requests. By recursively attempting ingoing connection attempts to all endpoints observed in the exchange of ADDR messages, it is possible to spider through the subset of nodes forming the backbone network of contactable peers. We use the data derived from one such public crawler^16^ and its MaxMind legacy GeoIP database to geolocate all currently contactable Bitcoin peers, which typically number between 5000 and 6000 nodes, and plot them on the Google Data Arts Team's open platform “WebGL-Globe”.

Using data from *Blockchain.info* (which provides transaction messages, including the IP address of the first peer that the Blockchain.info supernode is aware to have relayed the transaction), we then increment the columnar representation corresponding to the particular IP address by one unit to indicate the transactional activity.

We have found that this visualization greatly aids in the lay explanation of a peer-to-peer overlay network and the global nature of Bitcoin infrastructure and its activity. In this case, however, the transactional insight the visualization provides is of limited value since it is dependent on the particular latencies and connections of the Blockchain.info supernode. With the addition of topological data derived from Miller et al.^14^ and timing data from multiple triangulation nodes, it could prove a useful tool for monitoring network robustness and threats in real time.

## Analysis of a Denial of Service Attack

While conducting this work and exploring the *mempool* on a daily basis over the summer of 2015, a sustained attack upon the Bitcoin network became immediately visible and warranted further investigation:

A long-running source of disagreement within the Bitcoin community is the arbitrary 1 MB limit on the size of a block. Originally implemented to prevent certain denial of service attacks, it prevents the system from scaling beyond a transaction rate of only around four transactions per second. In 2015, unknown actors took it upon themselves to automatically generate economically insignificant spam transactions, in an effort to artificially increase the data rate and seemingly press home the need to raise the 1 MB limit. By visualizing these transactions mined into blocks over that period, it is possible to make several observations of interest.

**FIG. 7.**
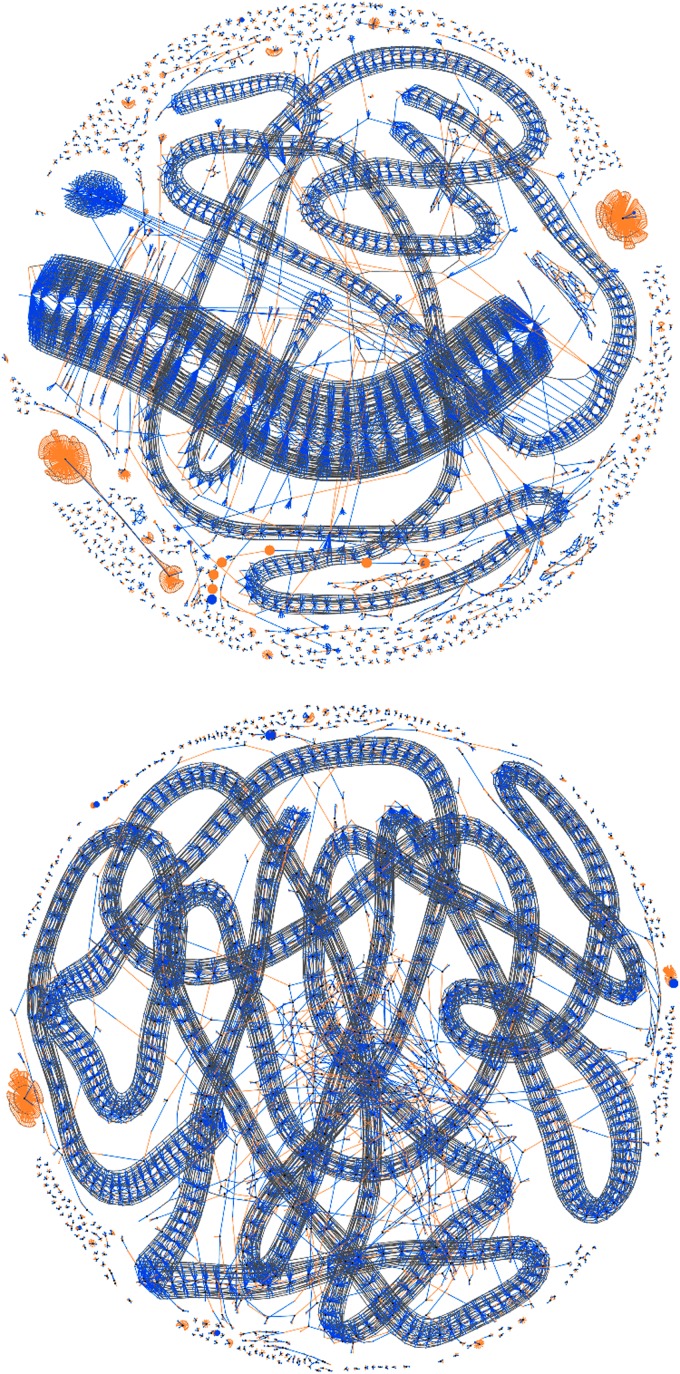
Blocks#364133,364618: Initial “parasitic worm” transaction rate attack.

The attack started with a sudden increase in the transaction rate with the formation of “parasitic worm” structures in the visualization due to the algorithmic high-frequency division of Bitcoin into tiny amounts to the same set of addresses, shown in Figure 7.

**FIG. 8.**
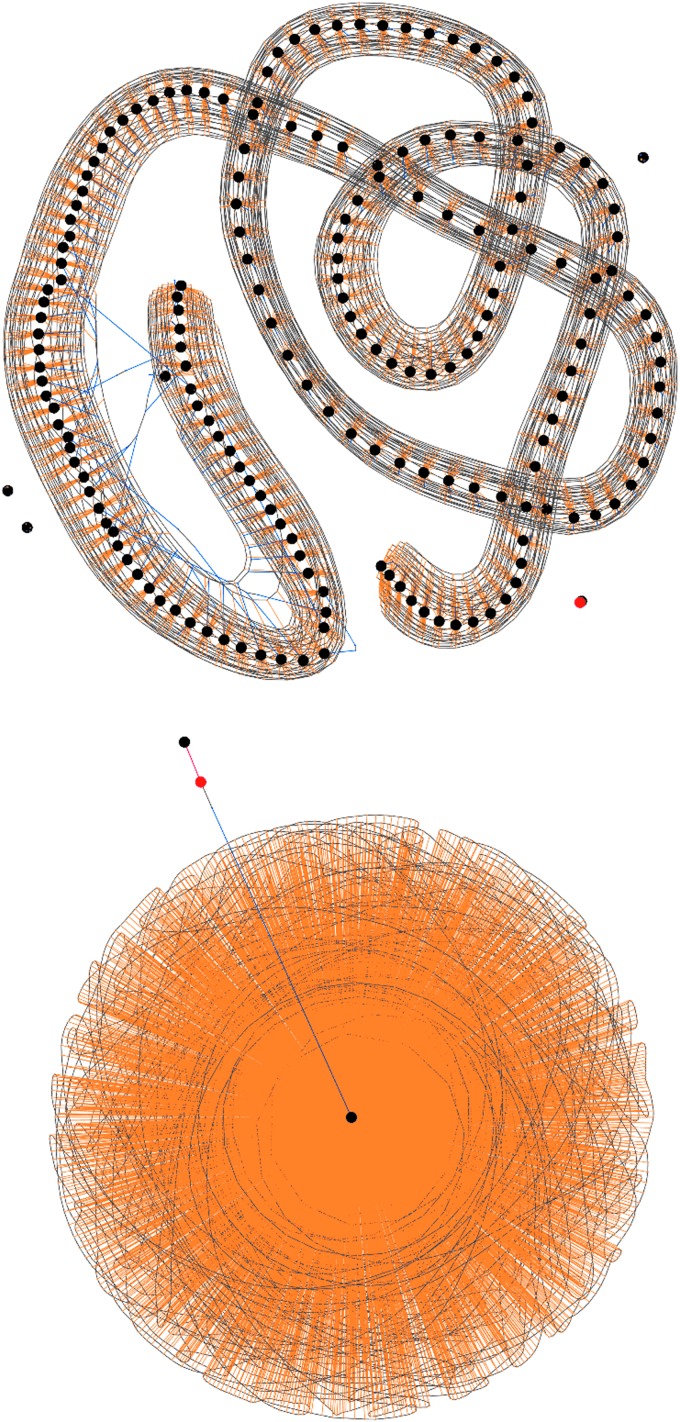
Blocks#364281,364292: Initial algorithmic responses to spam, the lower block showing the largest possible transaction.

Processing this volume of transactions occupied network resources and caused a degradation in the service of regular transactions. The attack's effects were amplified by the use of addresses with very low entropy dictionary private keys such as “cat” or “password1”. Similar in nature to throwing a handful of dollar bills into a crowded room, we quickly observed the algorithmic scramble to collect these multiple small amounts of Bitcoin, including the mining of the largest possible single transaction at 1 MB in Figure 8.

**FIG. 9.**
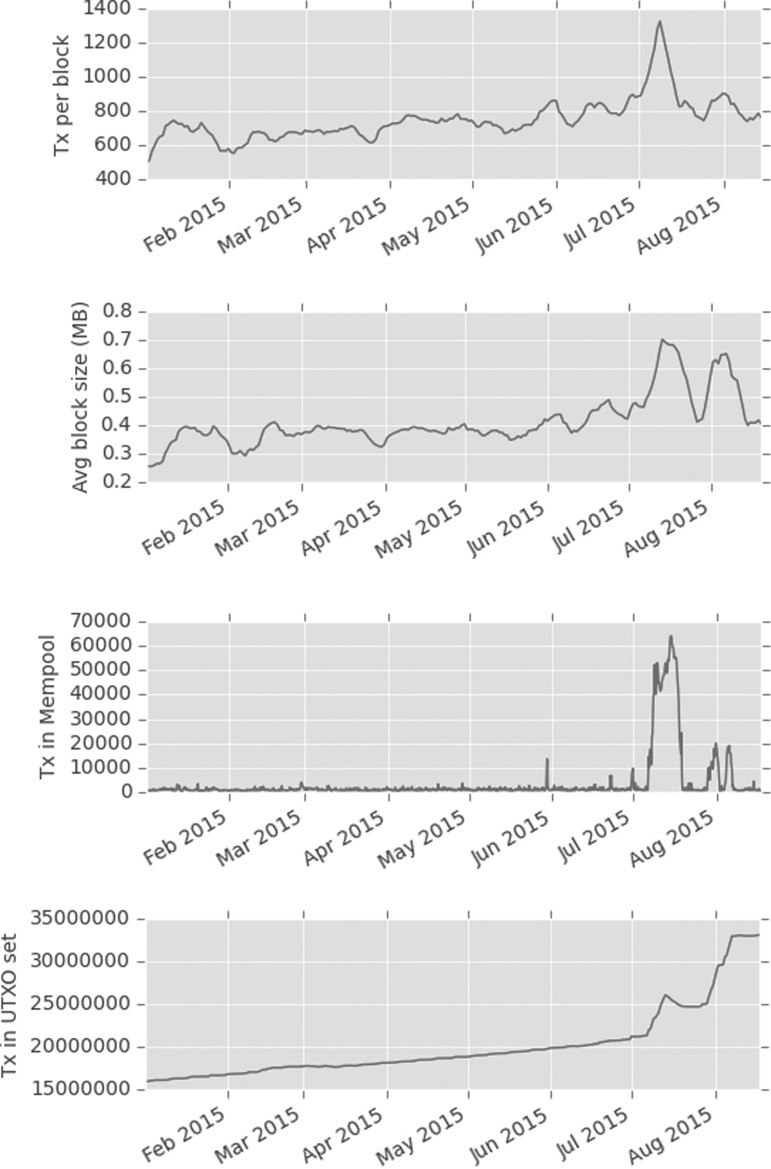
Network statistics showing the change from a transaction rate attack to the two-phased data density attack.

This transaction rate attack forming the parasitic worm structures persisted across many blocks. It caused delays in the processing of all transactions and a backlog of transactions in the mempool pending verification. However, even after the transaction rate returned to normal, it was evident that the network was still under duress. Figure 9 shows the sudden single increase in transaction rate, but only on inspection of the average block size does it become apparent that a second attack occurred in quick succession, the nature of which was data density rather than transaction rate.

**FIG. 10.**
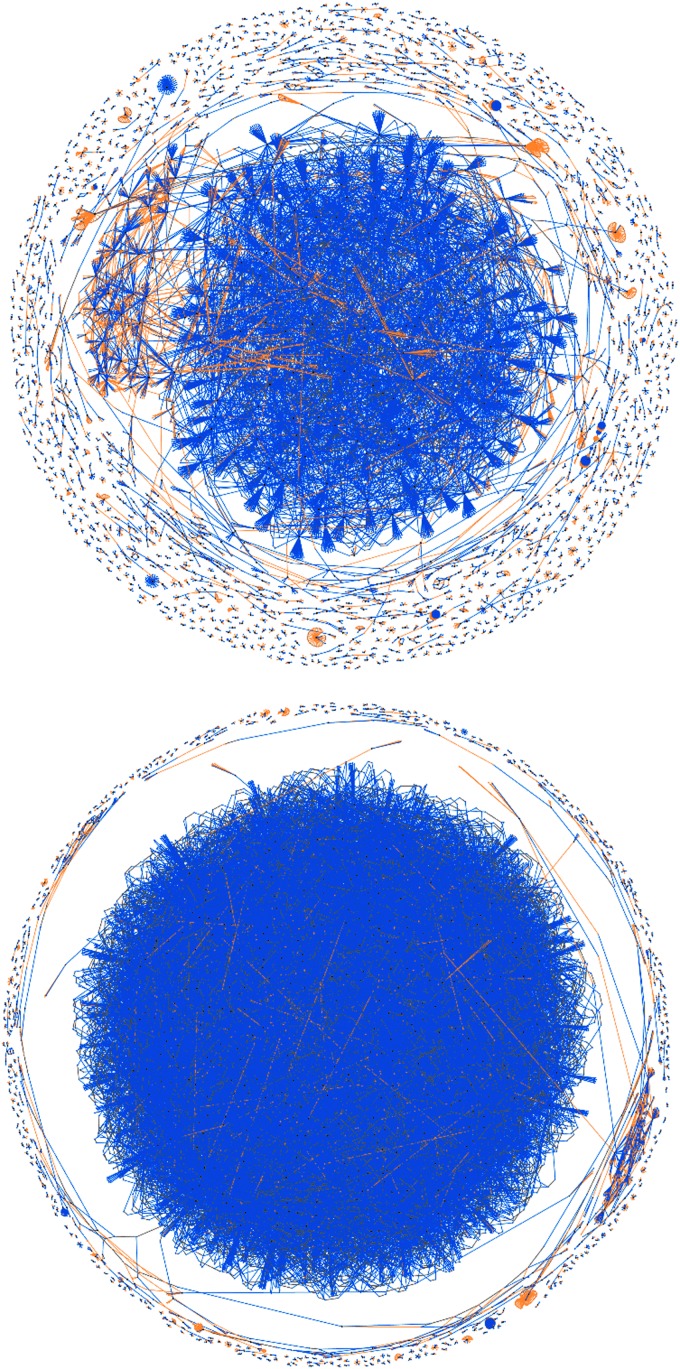
Blocks#367409,368580 from 29th July to 6th August 2015 show two distinct phases of the second data density-based “tumor” attack, note obvious change in algorithm parameterization to increase density.

This second attack occurred in two phases as shown by the change in gradient of the number of records in the *UTXO* set in Figure 9. The attack had a limited impact on the backlog of transactions in the mempool, but a very pernicious effect on the number of UTXOs. By studying the block visualizations over this period, we can see that a very different algorithm was used, generating a “cancerous tumor” structure. This attack is very much one of data density rather than transaction rate and probably conducted by an entirely separate second party. It is also obvious to note the point at which a simple constant parameter in the algorithm was amended to increase the data density of this attack in its second phase, shown in Figure 10.

Many of these insights arose from collaborative discussions among multidisciplinary researchers within the immersive visualization environment of the data observatory, which allowed the details of these visualizations to be interrogated as a group.

## Benefits of High-Resolution Visualization of the Bitcoin System

At current transaction rates, each block visualization typically contains a minimum totaling 5000 vertices (although it is not rare to get >20,000 in busy periods). This is where the advantage of rendering into a high-definition large-scale observatory proves its worth. Not only is the human visual system able to easily discern the associated patterns of behavior observable in the data but one can also physically approach the detail in the data and conduct a fine-grained analysis of one particular anomaly, while maintaining the context of the whole picture. Crucially, conducting these investigative discussions as part of a team of collaborators has been found to be most useful, especially when able to simply turn one's head to make comparative observations across multiple blocks simultaneously.

The graph visualizations described in this article maintain only minimal utility on a desktop screen during periods where the number of vertices increases beyond 10,000. Such periods in fact occur frequently, for instance after a long delay between the mining of blocks or of a massively increased transaction rate due to artificial network stress.

In our case, this high-resolution large-scale visualization has proven to have some additional benefits:

• Introducing the whole Bitcoin system to the general public, given its abstract nature, is no simple task. By exposing all of the system's tightly coupled components on the display at once, explanation and group discussion have been greatly facilitated. The visualizations have also shown their educational worth having been used on national television^17^ to materialize some of the abstract concepts of Bitcoin and explain associated blockchain technologies.
• High-frequency algorithmic behaviors previously hidden in the dense data set became immediately obvious and differentiable, greatly accelerating further initial investigation employing machine learning/pattern recognition techniques.
• Collaborative academic discussion of the Bitcoin system and its observed behaviors within the Observatory space among both lay practitioners and experts has enriched researcher's decision-making processes as to where to concentrate their efforts.

Given the nature of the Bitcoin data set described above, we do not doubt that these additional benefits would largely be absent without the high pixel density canvas and exploratory space afforded by the big data visualization tool presented in this study. We also believe that these benefits are transferable to other big data problems.

## Evaluation of the Effectiveness of the Bitcoin Visualization

To determine the effectiveness of this visualization of the Bitcoin system, observations were made on the various visiting groups to the Data Observatory, totaling over 900 people. Among the general public were visiting executives from companies, visiting researchers in various fields, as well as researchers from departments based at Imperial College.

Almost all visitors had heard of Bitcoin and recognized it as a currency. Aided by the peer visualization, almost all visitors recognized the mempool visualization as representing all global transactions, rather than a limited subset. Upon explanation of the visual representation of a transaction, they were able to understand the layout of the linking between transactions far more clearly than the raw data, and the majority of people were then able to spot anomalous patterns in the visualization and question their significance based on oral feedback after the initial presentation.

For visiting executives, the conversations tended toward questioning the anonymity of the data to ascertain the feasibility of tracking transactions across time to determine their origin. They were able to identify the majority of formed structures, although generally were more interested in the ability to apply the visualization to alternative financial transactions.

For researchers from different fields, a large number of observations were made about the resemblance to areas in their areas of expertise. In particular, those in medical and biological fields made reference to the visual similarities between the network attacks and parasitic organisms. Again, there was ease in the recognition of structures as well as the ability to identify them in further block illustrations.

The greatest benefit, however, was to researchers both internal and external specifically working in the field of cryptocurrencies. As with previous groups, the large size of the visualization allowed viewing as a group rather than an individual, but in addition, the ability to identify an individual transaction in a block that might contain several thousand. This can be then recorded for later study or investigated within the space. The ability to identify large transactions, as well as identify the patterns for hostile algorithms, coin-tumbling services, payment services, and otherwise unknown transaction patterns allowed for continuing research.

## Conclusions

This article presents the development of tools to gain an exploratory understanding of associated patterns of behavior in the densely connected dataset of all Bitcoin transactions. Compared to previous bottom-up approaches exploring data from singular source transactions, our approach has been to generate a top-down system-wide visualization enabling pattern detection subsequently allowing drilled-down detail into any transaction. Furthermore, we have shown how we combine both the transaction and address graphs into one high-fidelity visualization of associations.

Precisely, these visualizations have elegantly revealed the structure of the recurring high-frequency patterns of an algorithmic denial of service attack on the Bitcoin system and revealed previously hidden insights into the multiple distinct phases of such attack. Identification and classification of such observable patterns of behavior among other recurring patterns such as money laundering have provided useful kernels for analysis and discussion among multidisciplinary researchers.

In brief, the described visualizations have proven their usefulness for three distinct purposes: (1) understanding transaction patterns, (2) collaboratively evaluating and exploring these patterns with groups of experts, and (3) providing an introductory educational primer on the operation of the Bitcoin system to the general public.

## Abbreviation Used

UTXO: unspent transaction output
BTC: bitcoin

## Notes

^*^ A low resolution video demonstrating the system can be found at: https://imperialcollegelondon.app.box.com/v/bitcoinVis
